# The Role of the Microbiome in Oral Squamous Cell Carcinoma with Insight into the Microbiome–Treatment Axis

**DOI:** 10.3390/ijms21218061

**Published:** 2020-10-29

**Authors:** Amel Sami, Imad Elimairi, Catherine Stanton, R. Paul Ross, C. Anthony Ryan

**Affiliations:** 1 APC Microbiome Ireland, School of Microbiology, University College Cork, Cork T12 YN60, Ireland; amelsami1@hotmail.com (A.S.); catherine.stanton@teagasc.ie (C.S.); p.ross@ucc.ie (R.P.R.); 2 Department of Oral and Maxillofacial Surgery, Faculty of Dentistry, National Ribat University, Nile Street, Khartoum 1111, Sudan; 3 Teagasc Food Research Centre, Moorepark, Fermoy, Cork P61 C996, Ireland; 4 Department of Paediatrics and Child Health, University College Cork, Cork T12 DFK4, Ireland; tonyryan007@gmail.com

**Keywords:** oral microbiome, gut microbiome, oral squamous cell carcinoma, head and neck cancer, microbial

## Abstract

Oral squamous cell carcinoma (OSCC) is one of the leading presentations of head and neck cancer (HNC). The first part of this review will describe the highlights of the oral microbiome in health and normal development while demonstrating how both the oral and gut microbiome can map OSCC development, progression, treatment and the potential side effects associated with its management. We then scope the dynamics of the various microorganisms of the oral cavity, including bacteria, mycoplasma, fungi, archaea and viruses, and describe the characteristic roles they may play in OSCC development. We also highlight how the human immunodeficiency viruses (HIV) may impinge on the host microbiome and increase the burden of oral premalignant lesions and OSCC in patients with HIV. Finally, we summarise current insights into the microbiome–treatment axis pertaining to OSCC, and show how the microbiome is affected by radiotherapy, chemotherapy, immunotherapy and also how these therapies are affected by the state of the microbiome, potentially determining the success or failure of some of these treatments.

## 1. Introduction

In the Global Cancer report 2018, head and neck cancer is thought to be the eighth most commonly occurring cancer in the world [1], with oral squamous cell carcinoma being the most common presentation. According to The International Classification of Diseases for Oncology, World Health Organisation, oral cavity cancer includes the locations of the inner lip (C00.3–C00.9), the tongue, excluding lingual tonsil (C02), the gum (C03), the floor of the mouth (C04), the palate (C05), and other or unspecified parts of the mouth (C06) [2].

Although, in some parts of the world, there is a decreasing trend of oral squamous cell carcinoma (substituted, in part, by the increasing trend of human papilloma virus-associated oropharyngeal cancer) [3], incidence of the disease is very much still relevant all over the world. In Africa, oral squamous cell carcinoma statistics are highly underreported, but, in a review by Faggons et al., 2015, the smokeless tobacco products Toombak and Kola nut, used in Sudan and Western African countries, respectively, were the cause of the greatest number of head and neck cancer cases on the continent [4]. Meanwhile, South East and Central Asia accounts for almost half of oral squamous cell carcinoma cases globally [3], while, in the United Kingdom (up to 2016), there were 52,829 oral cavity cancer cases reported. In the Republic of Ireland, oral cancer cases are said to be significantly increased in both genders, but particularly amongst women [2,5].

Tobacco (cigarette smoking and smokeless tobacco) and alcohol remain the most established risk factors for the development of oral squamous cell carcinoma. Betel quid is commonly used in Asia and is the cause for the high oral cancer burden there, while other known aetiological predispositions include low socioeconomic status, poor intake of fruit and vegetables, poor oral health, genetic diseases associated with DNA repair and chronic inflammation.

Indeed, on that latter note, chronic inflammation is thought to be the cause of one in four cancers [6]. How this may relate to the aetiopathogenesis of oral squamous cell carcinoma is an important undertaking. Compelling evidence now exists for the association of chronic periodontal disease and the development of head and neck cancer in general and oral squamous cell carcinoma specifically. For every millimetre of alveolar bone loss, a four-fold increased risk of developing squamous cell carcinoma of the head and neck cancer unfolds. Furthermore, those with periodontal disease are thought to be at greater risk of poorly differentiated cancers [7,8]. Since periodontal disease is a microbial disease, this sets the tone for the question: what is the role of microorganisms in the aetiology of oral squamous cell carcinoma? This review sets out to answer this question, delving into how both the oral and gut microbiome may sculpt, in many ways, the outcome of an oral squamous cell carcinoma from start to finish.

## 2. Role and Function of the Microbiome

The microbiome, including oral, is the genome of all microorganisms, their interactions and their products which include bacteria but also viruses, archaea and fungi [9,10]. The “bacterial” microbiome resides in the lumen of the gut and the oral cavity; however, the skin and vagina also harbour a diverse microbiome. Bacteria in the gut reside either on the epithelium or pass through the lumen, both offering important yet different roles in maintaining homeostasis between the host and the microbiome and have the distinct ability, from an early age, to sculpt the systemic immune response, whereby constant interactions between the gut microbiome and the mucosa regulate inflammation and mediate immune tolerance through bacterial translocation [11,12]. Commensal bacteria help sustain a healthy immune state throughout the whole body, serving to educate the various stages of development of different immune cells that later become necessary in the fight against cancer cells, including oral squamous cell carcinoma (OSCC) [13]. The functions of the gut microbiome include the metabolism of diet components and vitamin production, as well as release of short chain fatty acids, microbial metabolites that further promote anti-inflammatory responses such as the release of interleukin (IL) 10, the inhibition of IL-6, tumour necrosis factor alpha (TNF) [14] and the activation of G protein-coupled receptors, present on epithelial cells as well as immune cells, and have a wide range of immune-inflammatory responses, including tumour cell deactivation [15,16].

In the oral cavity, the normal microbiota colonise space, preventing pathogenic microbes from establishing themselves as well as exerting healthy microbial antagonism and quorum sensing. Meanwhile, the host immune system is quite successful in maintaining a tolerant state, mainly through the function of mucosal dendritic cells and the secretion of anti-inflammatory cytokines such as IL-10, and prostaglandin E2, which suppress the immune system through the formation of T regulatory cells [17], thus displaying an intricate and well-ordered harmony of stability between oral mucosal dental tissue health and the different microorganisms’ requirements for survival. It is only when the environment starts to favour shifts towards a dysbiotic picture that pathogenic bacteria begin to act at the forefront, unravelling their roles in various oral diseases.

## 3. Understanding the Microbiome First in Health, Then in Disease

**Figure 1:**
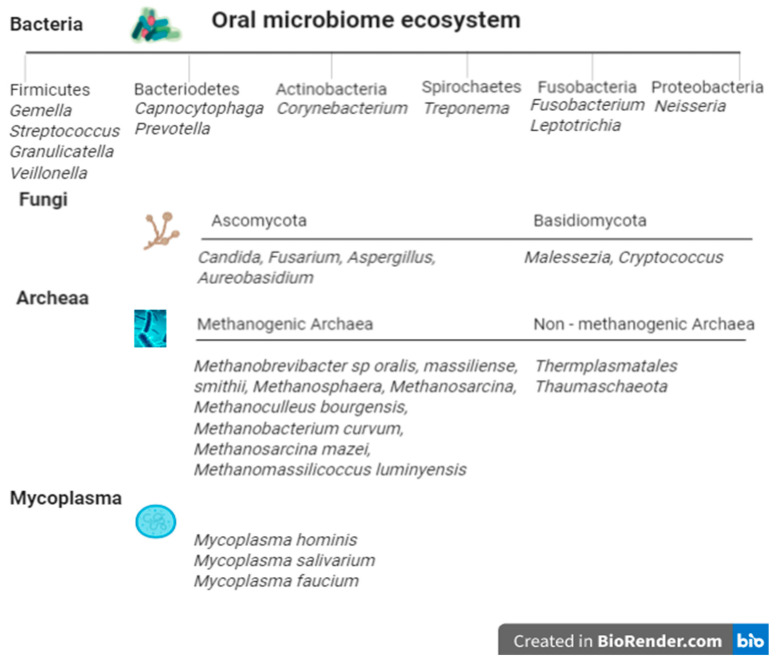
The oral microbiome ecosystem classification. References: [9,10,20,21].

In 2010, Delwhirst et al. assembled the Human Oral Microbiome project, identifying 619 bacterial taxa in 13 phyla, utilising 16S rRNA gene sequencing technology [9]. Figure 1 depicts the classification of the oral microbiome ecosystem in health. The most abundant genera of the oral cavity are noted with regard to their respective phyla, while archaea are recognised as either methanogenic or non-methanogenic forms and have been isolated predominantly from subgingival pockets. Fungal genera are also described according to their respective divisions; however, 30% of fungal communities remain largely unculturable. Protozoa such as *Entamoeba gingivalis* have been identified from within the oral cavity [18], while the commonest mycoplasma include *Mycoplasma salivarium* and *orale* [19] and are regarded as parasitic organisms.

A large part of the oral microbiome or “core” microbiome is comparable between individuals; however, every individual also has unique factors (age, behavioural habits, oral health especially saliva, immune status) that can change and modify the microbiome over time. *Bacteroidetes* (mainly the genus *Prevotella*), as well as the *Veillonella* genus, are found to increase in abundance with age, while others such as *Granulicatella* become decreased [22]. Pre-tooth eruption years predominantly display aerobic species such as *Streptococcus salivarius* while post-tooth eruption exhibits a microbiome that is much more diverse with new inhabitants to the hard surfaces of teeth and gingival crevices, particularly anaerobic bacteria. Iatrogenic environmental changes such as the placement of restorations, orthodontic appliances, implants and dentures can all place a map change in the oral microbiome. *Staphylococcus* species have been shown to have a high affinity for the titanium of implants [23], while those with orthodontic appliances have increased *Streptococcus mutans* and *Lactobacillus* species [24]. In a study by Si et al., 2016, the subspecies *Fusobacterium nucleatum animalis* was found to exclusively colonise the dentures of patients with denture stomatitis. This subspecies has been associated with pathogenic disease, including colorectal cancer, cardiac disease and stillbirth [25].

Neonates born by vaginal delivery, those delivered full term and who undergo breastfeeding are generally in the landscape of a healthy microbiome development, with a much more diverse and stable microbiome profile, while those who are born by Caesarean section, delivered premature, provided with formula milk or exposed to antibiotics early in life generally favour a more dysbiotic course of microbiome development [15,26].

Indeed, any dysbiosis that occurs in the gut microbiome has been associated with many diseases that include inflammatory bowel diseases, type 2 diabetes, atherosclerosis, obesity, mental disorders and cancers of the gastrointestinal tract, as well as lung, breast and haematogenic malignancies [27,28].

Importantly, the oral microbiome is an important factor to consider not just in the pathogenesis of OSCC, but also in non-head and neck-related cancers. Oral bacteria have been implicated in oesophageal squamous cell carcinoma and adenocarcinoma (*Porphyromonas gingivalis* and *Tannerella forsythia*, colorectal cancer (*Treponema denticola, Prevotella intermedia*) and pancreatic cancer (*Actinobacillus Actinomycetemcomitans* and *Porphyromonas gingivalis*) [29].

Bacterial communities of a healthy person and those of an OSCC patient have been shown to differ considerably with regard to function, such that those bacterial genes involved in methane metabolism, oxidative phosphorylation and carbon fixation are enriched in the OSCC microbiome, while those involved in amino acid metabolism and folate biosynthesis are not. [30]. Some members of the microbiome are regarded as “key players”, whereby, even in low abundance, their effect is powerful enough to impact on the remaining environment [31]. A richer and diverse microbiome with a higher number of reads and operational taxonomic units is generally well recognised in healthy persons, while edentulousness and disease affiliations have been associated with reduced richness and low diversity in the microbiome [32]. Guerrero-Preston et al., 2016, highlighted this phenomenon in OSCC cases specifically, where the total number of bacterial operational taxonomic units amongst OSCC samples was 3659, compared to that of healthy individuals (13,849) [33].

## 4. Microbiome and Pathogenetic Mechanisms Underlying OSCC

Ever since Virchow in 1863, the link between “bacteria, inflammation and cancer” progression has been put forward [34]. A statement more applicable to the present day would include how the oral microbiome, the epithelial barrier, the immune system and chronic inflammation exist in an elaborate oncogenic parallelogram [35]. A background of inflammation in relation to cancer development occurs in the context of infections, autoimmune disease and chronic and low-grade stimuli, such as from continuous irritation [36]. The oral microbiome not only drives the chronic inflammation that may precede OSCC, but is also involved in the direct influence on the host cell response [37]. Yost et al., 2018, showed that *Fusobacteria* had the highest number of transcripts or active genes in OSCC sites than any other bacteria, followed by *Selenomonas* and *Prevotella*, while *Bacillales, Gemella* and *Neisseria* evidenced more activity in healthy sites. Metatranscriptomic analysis of the OSCC-associated microbiome or “oncobiome” has shown the specific activities that are undertaken by these bacteria such as increased metal ion transport and nitrous oxide reductase, as well as tryptophanase and protease activity [38]. Increased ion transport around cancer sites is associated with catalysing radical ions and the promotion of cancer cell growth [39,40]. Other functional activities of the OSCC-associated microbiome include anaerobic respiration, proteolysis and the response against oxidative stress or radical species damage [41].

**Figure 2:**
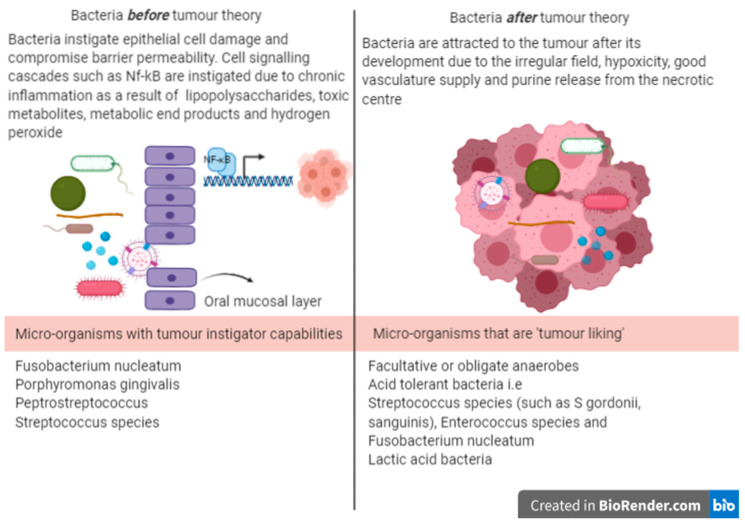
The possible connections between bacteria and development of oral squamous cell carcinoma (OSCC).

To provide lucidity in the relationship between the oral microbiome and OSCC, the “who came first” concept is discussed and illustrated in Figure 2. Here, two broad theories are formulated, in that either a single or group of bacteria may be the direct causative or driving factor in the pathogenesis of OSCC or may have the ability to restructure the microbiome to a more oncobiomic environment (bacteria before tumour) that damages healthy epithelial cells, or that bacterial presence in the OSCC tumour environment is opportunistic and is established after tumour development (bacteria after tumour). In either concept, nevertheless, it is the metagenomic and transcriptomic events which occur in the oral microbiome, that influence the change in oral cells and in conjunction with the host cell and immune response, these all have the final say in OSCC development. Furthermore, it is highly likely that a combination of both these theories occurs throughout OSCC progression.

In the bacteria after tumour theory, there are many factors that facilitate the attraction of bacteria to a tumour, including the irregular geographical anatomy and increased vasculature of a cancerous lesion, the hypoxic nature of the tumour environment that particularly favours facultative or obligate anaerobic, saccharolytic and acid-tolerant bacterial genera, the reduced host immunity at the tumour site, the presence of purines in the necrotic centre of the tumour, which can serve as nutrients for bacterial growth and cancer cell chemoattraction, as well as adhesion (through cell membrane glycoconjugates) to bacteria [42]. Some of the commonest Phyla associated with the OSCC environment include *Actinobacteria, Bacteroidetes, Firmicutes, Fusobacteria*, and *Proteobacteria* [43].

These arriving bacteria then reciprocate a synergistic relationship at the cancer site, indirectly promoting the disadvantage of tumour growth and progression, similar to the red complex associated with periodontal disease [44,45]. *Oribacterium* for example, are known to produce acetate as a metabolic byproduct, a substrate that is quickly utilised by hypoxic growing OSCC tumours [46].

In the bacteria before tumour theory, the oral mucosa can release numerous inflammatory mediators in response to pathogen-associated molecular patterns of bacteria that include lipopolysaccharides, polysaccharides, peptidoglycans, flagella and microbial DNA and RNA. These activate the host pattern recognition receptor molecules that include toll-like, lectin, nod-like and retinoic acid-inducible gene I-like receptors. Toll-like receptors (TLR) are the most prominent pathogen recognition receptors and can be classified as cell surface or intracellular forms. The cell surface types include TLR4 and TLR5, which recognise bacterial lipopolysaccharides of Gram-negative bacteria and bacterial flagellin, respectively, while intracellular TLRs generally recognise bacterial nucleic acids [47].

The activation of TLRs by bacteria predominantly triggers the MyD88 pathway, which culminates in the activation of the transcription factor; nuclear factor of kappa beta (NF-kb) and mitogen-activated kinase pathways (MAPK), responsible for the many proinflammatory pathways that sustain chronic inflammation [48]. Ultimately, this can eventually progress to haphazard oral cell proliferation and cytoskeletal abnormalities, as well as the inhibition of apoptosis of mutated cells that occurs via the modulation of the Bcl-2 family of proteins and the inactivation of retinoblastoma protein (pRb) as well as the activation of transcription factors such as STAT3 and NFAT [49].

Lipopolysaccharides (endotoxin forms of pathogen-associated molecular patterns in Gram-negative bacteria) also promote the release of IL-1, stimulating several pro-inflammatory cytokines such as IL-6 and TNF alpha, as well as vascular endothelial growth factor. The induction of matrix metalloproteinase (MMP) 9 through IL-1 can degrade the basement membrane and allow for an invasive and aggressive picture of OSCC [50]. The release of IL-6 further induces oxidative stress and can aid the adherence of cancer cells to endothelial cells by the upregulation of intracellular adhesion molecules. IL-6 also allows for the transcription of many anti-apoptotic gene pathways [37]. Bacteriocins and other proteins such as proteases (which can act like exotoxins) can stimulate host cells to release collagenases, prostaglandins and thromboxane, which further sustain mucosal damage, as well as degrading the immune products of the oral cell, or illicit the release of transforming growth factor B, which can cause abnormal proliferation in cells [51].

TNF alpha primarily exerts its effects through the nuclear factor of kappa B (NF-kB) pathway [52], observed to be upregulated in OSCC [53], and is a key pathway in bacterial-associated inflammation. Chronic levels of TNF alpha production have been shown to promote DNA damage by reactive oxygen species (ROS), and support tumour growth through angiogenesis [54]. ROS are involved in all stages of cancer development: initiation, promotion and progression. Thus, the oral microbiome may be an important source for unopposed oxidative stress in epithelial cells that may finally lead to unrepaired DNA mutations, cell division of these abnormally mutated cells and their eventual progression to metastasis [55].

Bacteria are also involved in the release of damaging metabolic end products such as volatile sulphur compounds, organic acids, and aldehydes, as well as the release of nitrosatable compounds. Acetaldehyde is a metabolic end product of many bacteria and has the ability to exert DNA damage and cause excess proliferation of the epithelium. Some of its producers include *Streptococcus* species (*S. gordonii, S. mitis, S. oralis, S. salivarius, S. sanguinis*), *Rothia* species, *Porphyromonas gingivalis* and other alcohol dehydrogenase-possessing organisms, including yeasts such as *Candida albicans* [56]. Through nitrosation pathways, bacteria can catalyse nitrosatable compounds to form N- nitroso compounds, which are associated with the development of cancers. This activity can also occur by fungi [57].

Hydrogen peroxide is another metabolic product that can diffuse passively into the epithelial cell, causing chromosome fragmentation and DNA damage such as double-stranded breaks, base modifications and DNA crosslinking. Hydrogen peroxide can also promote the release of oxidants that lead to post-translational modifications, especially in the P53 gene, tumour suppressor gene [6,35]. Hydrogen peroxide may also have oral cancer preventative pathways, and there is evidence in the existing literature to support the significantly higher expression of NLRP3 inflammasomes in OSCC cells that is associated with increased tumour sizes and lymph node metastasis [58], which oral bacterial hydrogen peroxide can suppress [59].

Volatile sulphur compounds, predominantly hydrogen sulphide and methyl mercaptan, are normally known to cause halitosis, and are produced by bacteria such as *Porphyromonas gingivalis, Prevotella intermedia* and *Fusobacterium nucleatum*, i.e., periodontal pathogens. These compounds have now been implicated in oxidative stress and DNA damage in oral cells. Even low levels of hydrogen sulphide can inhibit the enzyme superoxide dismutase, which is critical in preventing destructive ROS build up in human cells, while methyl mercaptan has been implicated in collagen breakdown, including type 4, and thus may have a role in OSCC invasion across the basement membrane [60]. Host proteins may also be metabolised or fermented into sulphides and nitrosamines by *Firmicutes* and *Bacteroides*, potentiating cell mutations [35].

## 5. Premalignant Lesions, the Story Before OSCC

There are various similarities and differences that exist in the microbiome environment of potentially malignant lesions such as leukoplakia, submucous fibrosis and lichen planus, as well as OSCC and other cancers. Lee et al., 2017, noted significant variations in the salivary microbiome of those with OSCC and oral premalignant lesions in the genera *Bacillus, Enterococcus, Parvimonas, Peptostreptococcus*, and *Slackia* [61]. Indeed, an interesting species is *Rothia mucaliginosa*, which was found to be abundant in leukoplakic lesions, but less so within OSCC lesions, indicating a possible role in early OSCC establishment [62]. An excellent producer but poor detoxifier of acetaldehyde, this species imparts significant oxidative stress in oral keratinocytes [63]. This species has also been found to be increased more so in those who use smokeless tobacco and may have a significant role in OSCC development, particularly in those with a high alcohol consumption.

Other authors have contrarily noted similarities between the microbiome of oral premalignant lesions and OSCC such as *Cloacibacillus, Gemmiger, Oscillospira*, and *Roseburia*, which were found to be abundant in both oral premalignant lesions and OSCC patients compared to controls [61], while similar microbial patterns have been observed between leukoplakic lesions and colorectal cancer, which include the abundance of *Fusobacterium, Campylobacter, Leptotrichia*, and *Rothia* species [64].

Taking lichen planus as an example, in a study by Choi et al., 2016, the genera *Leptotrichia* and *Acinetobacter* were increased amongst lichen planus cases, while *Streptococcus* was significantly decreased compared to controls. Notably, bacteria were found to be significantly more abundant in the basal layer and deeper lamina propria tissues of patients with oral lichen planus compared to controls, suggesting a bacterial causation in the liquefaction process associated with lichen planus histology. The rare finding of the intracellular bacterial infection of T cells was also noted and may explain the unique T cell infiltration that defines oral lichen planus [65] and provide a bacterial cause for why some lesions progress to OSCC.

## 6. Protumoural Bacterial Strains

Particular genera from the oral cavity that have been implicated in colorectal cancer development include *Fusobacterium, Peptostreptococcus, Porphyromonas* and *Parvimonas* [66]; thus, it is highly likely that such bacteria also have a role to play in the pathogenesis of OSCC. Examples of OSCC-associated genera include *Actinobacillus, Actinomyces, Aggregatibacter, Bacillus, Campylobacter, Catonella, Citrobacter, Clostridium, Dialister, Eikenella, Enterococcus, Exiguobacterium, Filifacter, Fusobacterium, Gemella, Haemophilus, Klebsiella, Lactobacillus, Micrococcus, Moraxella, Oribacterium, Parvimonas, Peptostreptococcus, Porphyromonas, Propionibacterium, Proteus, Pseudomonas, Rothia, Slackia, Staphylococcus, Streptococcus, Streptomyces* and *Tannerella* [33,61,67,68,69,70,71,72,73].

In a study by Sakamoto et al., 1999, the bacterial translocation of members of the oral microbiome, in particular of *Peptostreptococcus*, occurred from OSCC metastatic tumours to the cervical lymph nodes. This suggests that the oral microbiome may be directly implicated in the invading front [74] and may even be involved in metastatic events. The species *Peptostreptococcus stomatitis* has been isolated from both OSCC and colorectal cancer and is thought to contribute to the invasive tumour environment by the production of several acids such as acetic, butyric, and isovaleric acid [37].

A panel of bacterial species have been isolated from OSCC and include *Aggregatibacter segnis, Capnocytophaga gingivalis, Eubacterium saburreum Exiguobacterium oxidotolerans, Fusobacterium periodonticum, Fusobacterium nucleatum, Gemella haemolysans* and *morbillorum, Johnsonella ignava, Leptotrichia buccalis, Neisseria flava, Neisseria flavascens, Peptostreptococcus stomatitis, Porphyromonas gingivalis, Prevotella oris* and *melaninogenica, Pseudomonas aeruginosa, Staphylococcus aureus, Streptococcus gordonii, parasanguinis* and *salivarius* [43,49,67,70,75,76]. Rare species to inhabit the oral cavity, such as *Bacteroides fragilis* [77], previously unnamed bacteria such as *Actinomyces* (*oraltaxon_170*) and *Streptococcus* (*oral taxon 058*) [70,78] and the environmental species *Dietzia psychralcaliphila* and *Gordonia sputi*, have all also been identified from OSCC samples [68]. Some of these species have been researched in more detail, such as *Fusobacterium nucleatum* and *Porphyromonas gingivalis*, which will be discussed in more detail; however, most have not been pondered in relation to both their singleton and polymicrobial roles in the OSCC-associated microbiome.

*Pseudomonas aeruginosa* is a rare species to be isolated from OSCC, but has been implicated in carcinogenesis due to its ability to cause DNA breakage in epithelial cells [79] as well as having the ability to promote invasion and metastatic change. The bacterium promotes inflammation via its flagella, lipopolysaccharides, and ExoU cytotoxin, which can activate the NF-kB pathway and cause IL-8 secretion [80].

Perhaps the most “award winning” species that have been associated with OSCC development are the Gram-negative, anaerobic and invasive bacteria *Fusobacterium nucleatum* and *Porphyromonas gingivalis*, both of which have been shown to induce OSCC in a mouse model [81]. These two species have also been to date, robustly linked with gastrointestinal cancers [82,83]. Although *Fusobacterium nucleatum* is also related to healthy sites of the oral cavity, it has major roles to play in several oral diseases that include chronic and aggressive periodontal disease and endodontic infections, as well as more recently being implicated in OSCC development [84]. *Fusobacterium nucleatum* has been associated with high-grade dysplasia, lymph node metastasis and poor cancer progression [85,86]. The *Fusobacterium nucleatum* subspecies *polymorphum* was found to be significantly increased in 20 OSCC biopsies in a study by ElHebshi et al., 2017 [79].

*Fusobacterium nucleatum* can induce the expression of B defensin 2 (an antimicrobial peptide) from oral epithelial cells, which causes the release of potent pro-inflammatory cytokines such as IL-6 and IL-8 [85,87]. Increased B defensin 2 has been implicated in the pathogenesis of the potentially malignant disorder oral lichen planus, as well as OSCC of the tongue [88]. Through B defensin 2, mast cell degranulation also occurs, causing histamine release that further stimulates B defensin production; thus, a perpetuating cycle of chronic inflammation is allowed to continue and become established with the release of proinflammatory cytokines, the recruitment of immune cells and progressive angiogenesis [88].

An adhesin of *Fusobacterium nucleatum*, FadA, can bind to E-cadherin on epithelial cells, deactivating it to promote mucosal permeability. In turn, free intracellular B catenin allows for increased transcription of Wnt, a transcription factor involved in increased cell proliferation. Fusobacterial proteins such as Fap 2 can block and downgrade natural killer and T cell activity by the binding of Fap 2 with the inhibitory T cell immunoreceptor [28]. Increased activity of MMP9 and MMP13 also occurs, which allows for tumour invasion and metastasis by the degradation of the basement membrane [89].

*Fusobacterium nucleatum* has been associated with a subtype of colorectal cancer that exhibits molecular gene silencing by CpG island methylation. In OSCC lesions with CpG island methylation, over expression of the immune checkpoint receptor programmed death-ligand 1 (PD-1) has also been shown and thus these tumours may be more sensitive to immune checkpoint blockade [90]. Whether OSCC lesions associated with *Fusobacterium nucleatum* abundance have a better prognosis if treated with drugs such as Pembrolizumab requires further research.

*Porphyromonas gingivalis*, on the other hand, has been identified as an independent and significant risk factor in cancer-related deaths in the oral cavity and throughout the remaining oral digestive tract, pharynx, oesophagus, stomach, pancreas, liver, and colon and rectum [91]. Higher levels of antibodies against *Porphyromonas gingivalis* infection have been associated with a much higher increased risk of pancreatic cancer [92]. *Porphyromonas gingivalis* has been isolated from non-periodontal disease locations such as the deep crypts of the tongue and the buccal mucosa and is becoming more and more entrenched in its unique and somewhat hidden role in OSCC development [93]. Katz et al. (2011) isolated *Porphyromonas gingivalis* at both higher and more intense levels from OSCC samples as well as from poorly differentiated OSCC cells; however, further research is required to understand if this bacteria has a specific and common role in the differentiation of OSCC tumours [69].

*Porphyromonas gingivalis* imparts much interference on the host response to oral cancer. The bacterium can activate and complement TLR 2 and 4 and causes the release of pro-inflammatory cytokines such as IL-8. *Porphyromonas gingivalis* works intracellularly to prevent cell apoptosis through the inhibition of cytochrome C, downregulation of caspase 3 activity, secretion of the anti–apoptotic enzyme nucleoside diphosphate kinase that causes the inhibition of the P2X receptors on epithelial cells and the upregulation of microRNA 203, with the subsequent result of apoptosis quelling [94,95] while also upregulating the anti-apoptotic genes Bcl-2 and survivin [28,96,97]. In a study by Geng et al. (2010), *Porphyromonas gingivalis* was found to be associated with the upregulation of colon cancer-associated transcript 1, a long, non-coding RNA that has been found to be upregulated in several cancers and has also been described in oral epithelial cells, as well as in the upregulation of the enzyme production; nicotinamide N methyltransferase, which has been associated with malignancy and cancer stem cells, and was also detected in OSCC patients [93]. Gingipains of *Porphyromonas gingivalis* can cleave and activate MMP9 to its mature form, which can degrade the basement membrane, aiding OSCC metastasis [98].

Furthermore, *Porphyromonas gingivalis* can help OSCC cells to bypass the immune system by activating PD-L1 to bind to its receptor, PD-1, mediating profound T cell inhibition [99], and can induce the expression of the B7-H1 and B7-DC receptors in OSCC cells that leads to the apoptosis of activated T cells [100]. *Porphyromonas gingivalis* has been shown to induce epithelial to mesenchymal transition and can accelerate the cell cycle by affecting the p53, PI3K and cyclin pathways [101], primarily through its FimA adhesin molecule [102]. The bacterium also downgrades the activity of plakophilin, a key molecule in stabilising epithelial cells, and thus can promote metastatic change [93]. The bacterium is also one of the major producers of the carcinogen acetaldehyde and has a role to play in promoting autophagy of cancer cells, promoting chemotherapeutic resistance [103].

Thus, these two species, *Fusobacterium nucleatum* and *Porphyromonas gingivalis*, both have considerable virulence characteristics that allow them to be significantly involved in OSCC progression. They also act in a synergistic fashion, whereby metabolic end products of *Fusobacterium nucleatum* help promote the growth and the pathogenic potential of *Porphyromonas gingivalis* [104,105].

Furthermore, an important concept in the relationship between the microbiome and OSCC is that of “bacterial trending”, portrayed by Mukherjee et al. (2017). Here, it is suggested that there are multiple different combinations of genera that exist in parallel, but may contribute to the same phenotypical outcome, that is, OSCC development. An example of these are the high *Rothia* and low *Fusobacterium* and high *Eikenella* and high *Fusobacterium* combinations [72]. This indicates that the oncobiomic communities of OSCC are quite dynamic and maybe even interchangeable, as well as being much more individualistic from person to person. Indeed, both intra- and interkingdom connectivity (bacterial–bacterial/ fungal–fungal and bacterial–fungal relationships) may be vital in the better understanding of OSCC pathogenesis. Such examples include the synergistic and mutualistic relationship between *Candida albicans* and *Streptococcus* species, the cross feeding between *Streptococcus gordonii* and *Fusobacterium nucleatum* [106] and the required presence of the latter bacteria in the survival of *Porphyromonas gingivalis*, as has been previously highlighted.

## 7. Protective Bacterial Strains Against OSCC Development and Progression

*Leptotrichia, Neisseria, Streptococcus mitis*, and *Haemophilus parainfluenza* have been shown by several authors to be absent or less abundant in OSCC sites [71,79]. The presence of *Corynebacterium* and *Kingella* in one study was associated with a decreased risk of developing head and neck cancer (HNC); their presence was suggested to be cancer protective due to their ability to metabolise various toxic compounds by xenobiotic biodegradation. Other members of the oral microbiome that have been associated with a reduced risk of OSCC development include *Neisseria sicca, Parvimonas micra* and *Streptococcus anginosus* [78,107] Indeed, *Streptococcus anginosis*, in particular, has been associated much more with oesophageal cancer rather than oral cancer [108].

## 8. Lactic Acid Bacteria, the Controversial Order

Lactic acid bacteria are a controversial issue in both pro- and anti-cancer progression arguments. These bacteria, commonly used in probiotic therapy, have shown success in the management of several disorders that include diarrhoea, allergy to foods, inflammatory diseases and, recently, cancers such as colorectal cancer [109]. Lactic acid bacteria have been shown to harbour anti-cancer growth properties, by promoting apoptosis, exhibit anti-oxidative protection to cells, increase immune cell numbers and improve tumour suppression gene expressions [109]. Lactic acid bacteria can help eliminate the damaging effects of substances in the body, as well as being capable of the promotion and regulation of both innate and adaptive immune responses in the prevention of tumourigenesis. Such examples include *Lactobacillus rhamnosus* GG, a strain thought to have the ability to lower the chronic inflammation that is linked with cancer development [110].

In the oral cavity, *Lactobacillus* species can inhibit the binding of other bacteria to epithelial cells or even arrest both bacterial and fungal growth [36]. They also have a role to play in improving the efficacy of drugs. The bacterial strain *Lactobacillus fermentum* has been shown to exhibit anti-inflammatory effects against oral cancer and may also work to modify or improve specific treatments against OSCC such as the TLR-9 agonists CpG oligodeoxynucleotides [111]. The addition of *Lactobacillus fermentum*-CQPC08 in a mouse with cancer of the tongue was found to increase serum G-CSF, GM -CSF, IgG and IgM, IL-4, IL-12, TNF-alpha, and IFN-gamma, as well as improving local tissue antioxidants and decreasing malondialdehyde—a damaging lipid peroxidation product—while better macrophage phagocytic ability was also observed [112]. In another study, *Lactobacillus plantarum* was found to induce apoptosis of oral cancer cells by the upregulation of PTEN and MAPK signalling and was thus proposed as a potential probiotic adjuvant in OSCC treatment [113].

On the other hand, lactic acid production by *Lactobacillus, Lactococcus, Leuconostoc, Pediococcus, Streptococcus* and *Peptostreptococcus* has been implicated in OSCC development [51]. Other acids produced by these bacteria include acetic, butyric, isobutyric, isovaleric, and isocaproic acids, which all reduce the environmental pH of an OSCC, contributing to cancer growth and spread. Furthermore, lactic acid itself can enhance gene transcription and DNA repair by the blocking of histone deacetylases [36]. *Lactobacillus*, in one study, was found to be increasingly abundant, relative to the increasing TNM staging of cancer patients [33,114]. Table 1 summarises both the possible protective and harmful roles associated with protumoural and protective bacterial strains in OSCC pathogenesis and progression.

## 9. Microbiome as a Potential Diagnostic Tool

The use of the microbiome as a diagnostic tool may be an important, non-invasive opportunity in the diagnosis of preclinical or early OSCC. Mager et al. (2005) described the species *Capnocytophaga gingivalis*, *Prevotella melaninogenica* and *Streptococcus mitis* as significantly elevated in the saliva of patients with OSCC with a diagnostic sensitivity and specificity of 80% and 82%, respectively [76]. In a study by Yang et al., 2018, an increased abundance of *Fusobacteria* was found in association with cancer staging, whereby those with Stage 1 and 4 OSCC had 4.35% and 7.92% abundances of this phylum, respectively. Of note in this study was the further decreased abundance of *Fusobacteria* amongst non-OSCC patients (2.98%) [30]. Thus, whether analysis of *Fusobacteria* levels in patients who are at risk of developing OSCC is a useful futuristic and non-invasive diagnostic tool may be of questionable interest. Indeed, the OSCC-associated microbiome may even be accurate enough to differentiate between OSCC and neighbouring cancers. The genera of *Actinomyces, Parvimonas, Selenomonas* and *Prevotella* were found to be in higher abundance in OSCC patients compared to oropharyngeal cancer patients [73].

## 10. Effects of Smoking, Smokeless Tobacco and Alcohol on the Oral Microbiome

The oral microbiome is affected by habits such as smoking, smokeless tobacco use and alcohol intake that can modify the oral mucosal ecological niche, allowing for modifications that may predispose a person to OSCC. Smoking is thought to shift the oral microbiome to a more “pathogenic rich” environment with enhanced pro-inflammatory virulence promotion [115]. Furthermore, commensal bacteria such as *Neisseria*, in particular, seem to be lost amongst smokers [114,116], while an abundance of *Fusobacteria*, *Mogibacterium* and *Tannerella* occurs [117]. Whether *Neisseria* has a protective effect on the oral mucosa requires further investigation; however, the loss of the proteobacteria *Neisseria* and *Haemophilus* is also a pattern amongst gastric cancer patients [118]. Nicotine has been shown to promote bacterial adherence to the oral mucosa [119], while the metabolite of nicotine, cotinine, can significantly increase epithelial cell invasion by *Porphyromonas gingivalis* [120]. Smoking has also been shown to reduce the host response to *Porphyromonas gingivalis* [121] and it is thought that the local immunosuppression caused by smoking can be one cause of a more novel and pathogenic microbiome picture [122].

Furthermore, bacteria play an important role in the increased activation of nitrosamines from different tobacco forms, including smokeless tobacco [123]. Indeed, smokeless tobacco products, in a study by Liu et al., 2016, were shown to promote OSCC-associated genera such as *Eubacterium, Streptococcus* and *Peptostreptococcus*, while tobacco-specific nitrosamines reduced commensal species such as *Veillonella*. Interestingly, in the same study, smokeless tobacco was found to limit the growth of *Fusobacterium nucleatum*, as well as *Eikenella corrodens* [124].

Meanwhile, the effects of alcohol consumption (> 1 unit per week), are also quite significant on the microbiome, with the enrichment of OSCC-associated bacteria such as *Campylobacter* species [64]. In another study, those whose alcohol intake was > 40 g/day, were shown to have significantly increased salivary acetaldehyde production while in combination with smoking, an alcohol consumption <30 g/day (females) and <40 g/day for males, increased salivary acetaldehyde by 100% [125]. Interestingly, while *Neisseria* are generally regarded as commensal organisms and are associated with an anti-OSCC picture, the genus is still involved in the significant production of acetaldehyde from ethanol. *Neisseria* genera were found to be increased amongst alcohol drinkers, suggesting that, among alcohol users, they have the ability to turn into commensal pathogens. Furthermore, both oral and gut *Lactobacillus* have been found to be decreased amongst heavy alcohol drinkers (>1–2 drinks/day) and amongst those who both smoke and drink. *Lactobacillus* are able to break down acetaldehyde and thus alcohol is directly implicated in the disruption of the microbiome, potentiating acetaldehyde-associated carcinogenesis [123,126].

## 11. Oral Mycoplasma

In a study by Henrich et al, 2014, It was found that *Mycoplasma salivarium* colonisation was significantly related to the surface of tongue OSCC in a patient with immunosuppressive disease [127]. Mycoplasma have been associated with the inhibition of the P53 tumour suppressor gene and the activation of the NF-kB pathway [128] in rodents. Interestingly, *Mycoplasma salivarium* has been detected by immunohistochemistry from epithelial cells of leukoplakic lesions [129] undergoing hyperplastic changes. Whether oral mycoplasma play a role in acting as immunomodulators in targeted persons, particularly those with immunosuppressive states, aiding OSCC progression, is yet to be investigated further.

## 12. Oral Archaea

Both methanogenic and non-methanogenic archaic forms have been isolated from the oral cavity. Archaea have been isolated from disease locations such as chronic and aggressive periodontal disease, apical periodontitis, recalcitrant chronic rhinosinusitis and peri-implantitis [130,131]. They can illicit both an IgG humoral immune response [132,133,134,135] as well as proinflammatory cytokine reactions that include TNF alpha and IL-1 [20], and thus may be a hidden cause of chronic inflammation in the oral cavity. Importantly, through interspecies hydrogen transfer, a harmonious syntrophic environment develops between archaea and many bacteria, including pathogenic periodontal forms and fermenting bacteria, such that the presence of oral archaea can indirectly support the growth of bacteria that may be involved in the pathogenesis of OSCC [136]. Furthermore, methanogenic archaea are also known to be capable of methylating heavy metals to volatile forms that are toxic to human cells [137], as well as the possibility that the carcinogen formaldehyde can be produced from methane. Increases in methanogenic archaea have been associated with colorectal cancer [138]; however, no literature exists with OSCC; thus, this is an important area for future research.

**Table 1:** Summary of possible associations of harmful and protective bacterial genera and strains and their association with OSCC development and progression.

**Type of bacteria**	**Genera/Strains**	**Possible associated roles in OSCC development and/or progression**
	*Actinobacillus*	Can upregulate the production of CCL20 in oral cancer cell lines [139]
**Harmful-associated (pro-tumoural strains) bacteria**	*Aggregatibacter*	Expression of proinflammatory cytokines [140]
	*Capnocytophaga*	May stimulate chronic inflammatory processes
	*C itrobacter*	In murine models, strains of Citrobacter induce mucosal hyperplasia and inflammation [141]
	*C lostridia*	Clostridial spores have been shown to have an affinity for hypoxic regions of solid tumours and may be factors in OSCC development Clostridial strains have however been used in cancer therapy [142]
	*Cutibacterium* (*Propionibacterium*)	Induction of pro-inflammatory cytokines (IL-6 and IL-8). Strains have been associated with prostate cancer [143]
	*E nterococcus*	Extracellular superoxide may produce genomic instability [144] Enterococcus strains associated with chronic inflammation and development of other cancers [145]
	*Eubacterium saburreum*	Periodate-resistant antigen (PS L13) [146] may elicit immune reactions
	*Exiguobacterium oxidotolerans*	High catalase activity may protect cancer cells [147]
	*Fusobacterium*	Stimulation of pro-inflammatory cytokines (IL-6, IL-8) FadA adhesin of *Fusobacterium nucleatum* can increase transcription of Wnt, causing increased cell proliferation Fap 2 reduces immune cell activity Increases matrix metalloproteinase activity, leading to degradation of basement membrane
	*G emella*	Associated with upregulation of interleukins (IL-23A) in cancer [148]
	*Johnsonella ignava*	May stimulate chronic inflammatory processes
	*Leptotrichia buccalis*	Contains a potent phenol-soluble lipopolysaccharide endotoxin that can stimulate inflammation [149]
	*Oribacterium*	Production of acetate as a metabolic byproduct, a substrate quickly utilised by hypoxic growing OSCC tumours
	*Parvimonas*	Associated with development of colorectal cancers [150]
	*Peptostreptococcus stomatitis*	Has been associated with invading OSCC front
	*Porphyromonas gingivalis*	Activation of complement system Pro-inflammatory cytokine release Prevents cell apoptosis (inhibition of cytochrome C, reduced caspase 3 activity and secretion of nucleoside diphosphate kinase) Further prevents apoptosis by upregulating anti-apoptotic genes BCL2 and surviving Upregulation of colon cancer-associated transcript 1 Increased enzyme production—nicotinamide N methyltransferase Gingipains activate matrix metalloproteinases Bacteria is a potent PD/1/PD- L1 activator, mediating immune bypass Induction of epithelial to mesenchymal transition FIMA adhesin molecule of bacteria affects cell cycle and alters tumour suppressor gene expression Acetaldehyde producer
	*P revotella*	Increased Activation of Toll-like receptor 2 and promotion of T helper type 17 cells and associated mucosal inflammation Potent activators of pro-inflammatory mediators IL-6 and IL-8 as well as TNF-gamma [151]
	*Pseudomonas aeruginosa*	Chronic inflammatory stimulation DNA cell damage May promote metastasis
	*R othia*	Potent acetaldehyde producers
	*Staphylococcus aureus*	Staphylococcal α-toxin can activate pro-inflammatory cytokines and activate nuclear factor-KB This bacterium has been found to be in increased abundance in squamous cell carcinoma of the skin (associated with actinic keratosis) [152]
	*Streptococcus gordonii, parasanguinis* and *salivarius*	Streptococcal strains can produce acetaldehyde and show alcohol dehydrogenase enzyme activity [153]
	*Tannerella* (*forsythia*)	Induces pro-inflammatory cytokine production Cysteine-like proteases may arrest cell cycle in G2 phase [154]
**Type of bacteria**	**Genera/Strains**	**Role in OSCC prevention and/or cessation**
**Protective bacteria**	*Streptococcus mitis*	Their presence is very important in the prevention of colonisation of more virulent micro-organisms [155]
	*Streptococcus gordonii*	May have protective role, such that epithelial cells do not respond to Porphyromonas gingivalis-induced epithelial cell proliferation stimulation [156]
	*Streptomyces*	Some species have been shown to inhibit cell growth [157], including apoptosis of cancer cell lines [158]
	*Neisseria*	Can break down toxins of tobacco
	*Corynebacterium* and *Kingella*	Can metabolise various toxic compounds by xenobiotic biodegradation
	*Veillonella*	Naturally inhabits the dorsum of the tongue and thus may have more protective properties in this region [159]

Lactic acid bacteria have both protumoural and protective effects. Protumoural factors include; lactic acid production that reduces the pH of the cancer environment, potentiating spread. Enhanced gene transcription alters DNA repair by the blocking of histone deacetylases. The protective factors of these bacteria include the binding and degrading of carcinogens, the production of anti-carcinogenic compounds [160], promotion of apoptosis (enhances MAPK activity), providing anti-oxidative (against reactive oxygen species (ROS)) protection to cells, increased immune cell numbers (T cells) and the induction of cytokines (INF-gamma, TNF-alpha) and improved tumour suppression gene expression [109].

## 13. Oral Mycobiome

The physical (adhesion and exclusion) and chemical (metabolic dependency and quorum-sensing) relationships between the mycobiome and bacteriome in the oral cavity are essential to maintain oral health. One of the limitations so far in microbiome research studies associated with OSCC is the continuous exclusion of fungal dynamics [31].

Similar to the bacterial microbiome, the richness and diversity of fungal species have been found to be significantly reduced amongst OSCC [161]. The absence of certain members of the oral mycobiome has been associated with HNC. *Malassezia* has been found to be increased in healthy individuals compared to cancer patients [162], with its presence found to be negatively correlated with Candidal presence [163], while, in a study by Shay et al., 2020, *Schizophyllum* was enriched in the samples who did not have HNC. This genus is thought to produce schizophylan, a polysaccharide compound with anti-cancer properties; thus, its absence in HNC patients may reflect the loss of its protective role [164]. In another study, the presence of *Emericella* was shown to be decreased in tongue cancer tissue compared to normal tissue. In colon cancer cells, *Emericella* is thought to increase p53 tumour suppressor expression and thus its absence in OSCC lesions of the tongue may have a role in allowing cancer development [72].

On the other hand, the fungal mycobiome has been implicated in both premalignant disorders and OSCC. *Candida albicans, dubliniensis, tropicalis, pintolopesii*, and *glabrata*, as well as *Saccharomyces cerevisiae*, have been isolated from chronic hyperplastic candidiasis, a premalignant lesion with a higher rate of severe dysplastic transformation [165]. Thus, increased oral Candidal presence has been associated with both oral epithelial dysplasia and its severity [166]. In a study by Perera et al., 2017, the genera *Candida, Hannaella*, and *Gibberella* were increased amongst OSCC lesions and *Candida albicans*, *Candida etchellsii*, and a fungus resembling *Hannaella luteola* were the enriched species in OSCC [161]. *Candida albicans* is also thought to impart a protective role at tumour sites for bacterial species such as *Porphyromonas gingivalis*; the implications of this bacteria in OSCC development are described earlier in this review [167]. Indeed, it is *Candida albicans* that has been the most extensively studied with regard to its role in OSCC development and progression. This species can colonise the epithelium by secreting aspartic proteases as well as stimulating epithelial cell endocytosis, which ultimately leads to keratinocyte cell damage. *Candida* species have been shown to produce N-nitrosobenzylmethylamine and acetaldehyde, both of which are potent carcinogens [165]. Furthermore, Candidal proteinases have been implicated in the degradation of the basement membrane protein laminin-332. Interestingly, this has been found only to occur at acidic pH [168] and thus the acidic nature of a tumour, promoted by certain bacteria, may play a synergistic role with *Candida* species in enhancing this effect.

## 14. Viruses

### 14.1. Human Papilloma Virus, Epstein–Barr Virus and Herpes Simplex

While all these viruses are known to cause various different forms of oral disease, they are generally not considered to play a role in OSCC development. The human papilloma virus is not considered to play a role in OSCC pathogenesis, as it is with oropharyngeal cancer, and is found in very low frequency amongst OSCC patients [169,170]; however, the Epstein–Barr virus is controversial. Although present in OSCC cells, this is thought to be due to normal clonal presence and continuous shedding [171,172]; however, a recent meta-analysis concluded that Epstein–Barr virus infection is in fact statistically associated with an increased risk of OSCC and that DNA regions such as Bam H1W, which is an oncogene, were detected in OSCC tissue [173]. In a large study of 155 OSCC samples from eight different countries and ethnicities, 55% of samples were positive for Epstein–Barr virus, while only 35% were positive for the human papilloma virus, with double infection occurring in 21% of samples [174]. However, there is still no clear evidence for a reproducible aetiopathogenetic link between Epstein–Barr virus and OSCC development.

Herpes simplex 1 is another virus associated with oral infection, but with no known association with OSCC development. However, antibody levels to herpes simplex viruses have been found to be higher amongst OSCC patients when compared with controls [175]. Interestingly, it has been shown that lactic acid bacteria and their bacteriocins can act as antiviral agents, particularly to herpes simplex virus. *Lactobacillus* species can prevent viral replication in host cells [176] and can suppress herpes simplex virus type 2 by lactic acid production in the vagina [177] and, as such, this may also be the case orally for herpes simplex type 1. Due to its toxicity to cancer cells, some authors have utilised attenuated forms of the herpes simplex virus in the treatment of OSCC, which was shown in mouse models to inhibit the growth of oral cancer cells [178]. However, there are several limitations to this form of cancer therapy—primarily the elimination of the virus from the cancer cells and the inhibition of the virus by the immune system [179].

### 14.2. The Prodigy of HIV and All Players of the Microbiome

While some authors advocate a similar oral bacterial microbiome between human immunodeficiency virus (HIV) patients and healthy individuals [180,181], others have found considerably reduced microbial diversity amongst HIV-positive persons, with increased abundance of the pathogenic genera *Megasphaera, Campylobacter, Veillonella* and *Prevotella*, and decreased abundance of commensal forms such as *Lactobacillus* and *Streptococcus* species [46,177]. Indeed, the gut microbiome in HIV infection resembles many other proinflammatory conditions such as inflammatory bowel disease of the gut [182].

A pathogenic microbial shift is associated with high plasma HIV RNA levels (*Veillonella* and *Prevotella*), while a commensal shift (*Neisseria* and *Haemophilus* species) is associated with antiretroviral therapy [46]. However, treatment of HIV still does not restore the microbiome to better health and increased abundance of the pathogenic *Campylobacter, Capnocytophaga, Fusobacterium, Prevotella*, and *Capnocytophaga* genera are still observed [183]. Specific species associated with the OSCC microbiome and its development have been particularly isolated from HIV individuals and include *Capnocytophaga* [180], *Prevotella melaninogenica* and *Rothia mucilaginosa* [46]. The latter species, is very much associated with leukoplakic lesions and the carcinogenic release of acetaldehyde [184] and thus it may have a role to play in the high risk associated with developing oral premalignant lesions and OSCC amongst HIV patients [185]. Interestingly, *Porphyromonas gingivalis* may be able to induce HIV reactivation through butyric acid production [186], while also mediating the epithelial cell entry of the virus [187], but whether this bacterium and virus have a synergistic role to play in OSCC development in HIV positive patients is a source for further investigation.

The mycobiome of those with HIV varied considerably compared to controls in a study by Mukherjee et al. (2014) [180]. Interestingly, in this study, the fungus *Pichia* was found only in non-HIV persons and was found to have considerable inhibitory properties in the growth of *Candida, Aspergillus* and *Fusarium* [180]. This would suggest that the oral candidiasis that often presents itself in HIV-infected persons may be, in part, due to the absence of the antagonistic role that *Pichia* plays in healthy persons.

## 15. The Microbiome–Treatment Axis in OSCC

During OSCC treatment, both the oral and gut microbiome are affected by many factors that include host diet, side effects of surgery and radiation, antibiotic administration, as well as local implications such as the development of oral mucositis and dry mouth [188]. A new concept in cancer treatment is pharmacomicrobiomics, that is, how the gut microbiome interacts with drugs, affecting the success or failure of a treatment. Through the use of probiotics, prebiotics and antibiotics, subsequent interventions may be carried out to gear up the microbiome for a successful interaction with a wide range of cancer therapies. Early stage OSCC is predominantly treated via a surgery and radiation-based approach, while, where used, chemotherapy is often in the form of the platinum-based drug cisplatin, which is used to radiosensitise oral cancer cells [189] or in more advanced case settings.

The oral microbiome has been shown to undergo significant changes during and after radiotherapy for OSCC [190]; however, the use of intensely modulated radiotherapy can help protect and stabilise the oral microbiome compared to conventional forms of radiation [191]. The side effects of radiation for OSCC include the development of mucositis, xerostomia, taste modification, dysphagia, osteoradionecrosis and trismus [192]. In radiation therapy, higher abundances of the cariogenic bacteria *Streptococcus mutans* and *Lactobacillus*, as well as *Staphylococcus, Enterococcus* and the fungus *Candida albicans*, parallel to a reduced presence of the commensal microorganism *Neisseria* [193,194], have been described. Indeed, *Lactobacillus* species may be increased up to 6 months post-radiation, as xerostomia is thought to create a favourable growth environment [195].

However, HNC patients who undergo radiation have been shown to have the distinct trend of increased *Candida albicans* counts and *Enterobacteriaceae* as part of their oral microbiome [196]. In comparison, the xerostomic microbiome of those with Sjogren’s syndrome was associated with only increased numbers of *Streptococcus mutans* [197]. Increased abundance of *Enterococcus* species in radiated patients may be associated with a high percentage of antibiotic resistance and may further contribute to infections of the oral cavity during OSCC treatment [198]. *Enterococcus* species can also enhance microbial proteolytic activity on fibronectin, further promoting mucosal damage and inflammation from radiation [104]. Radiation also has an inhibitory role in antimicrobial proteins that would normally keep *Candida* growth in check [162].

Soil genera such as *Derxia* and *Luteococcus*, the latter isolated from blood, have also been sequenced from patients with HNC undergoing radiation [199]. Interestingly, a significant decrease in abundance occurred post-radiation therapy for the OSCC-associated species *Prevotella melaninogenica* in those with HNC [190]. With relation to osteoradionecrosis, the most predominant species from affected mandibles were *Campylobacter gracilis, Fusobacterium nucleatum, Streptococcus intermedius, Peptostreptococcus*, and *Prevotella* species [200]. *Actinomyces israelii* has been significantly associated with the necrotic bone of this condition and is implicated in the chronicity and purulent characteristics [201].

Cancer chemotherapy has a great impact on both the oral and gut microbiome. With the former, this is thought to shift to more acid-tolerant bacteria, but still leading to a relatively commensal (*Lactobacillus* and *Streptococcus*) picture. [202]. Napenas et al., 2012, found a predominance of *Gemella haemolysans* and *Streptococcus mitis* species amongst patients undergoing chemotherapy; however, the most common genera identified from the oral microbiome during cancer chemotherapy include *Streptococcus, Staphylococcus, Klebsiella*, and *Enterobacter* [203].

The gut microbiome has a vital impact on the response to cancer chemotherapeutics and is involved by improving or compromising the efficacy of cancer drugs, as well as mediating their toxic effects. The microbiome has also recently been thought to play a role in chemotherapy-induced pain [204]. Locally, intra-tumour bacteria may also modulate chemotherapeutic effects [205].

The combination of *Lactobacillus* species (*Lactobacillus acidophilus*) and cisplatin was found to reduce tumour size and improve survival in a lung cancer mouse model [206]. The microbiome may also help upregulate proapoptotic pathways that improve the efficacy of cisplatin chemotherapy [207]. Other chemotherapeutic drugs used in advanced OSCC may also be affected by the microbiome. Gemcitabine is a synthetic pyrimidine antimetabolite that has efficacy in some cases of late stage OSCC [208]. *Gammaproteobacteria* have been shown to infer gemcitabine resistance, and thus co administration with ciprofloxacin can improve efficacy of this drug [205].

Oral mucositis has been associated with a marked loss of commensal bacteria such as *Actinomyces, Streptococcus, Granulicatella*, and *Veillonella* and an increased abundance of pathogenic bacteria such as *Enterobacteriaceae, Pseudomonas* and *Staphylococcus* species as well as *Escherichia coli*, *Fusobacterium nucleatum*, (in particular, the presence of *Fusobacterium nucleatum subspecies vincentii* in more severe mucositis), *Clostridiales, Treponema denticola* and *maltophilum, Porphyromonas gingivalis, Parvimonas micra* and *Prevotella oris* [207,209,210].

*Porphyromonas gingivalis, Candida glabrata*, and *Candida kefyr*, in a study by Laheij et al., 2012, were significantly associated with ulcerative oral mucositis [209]. Indeed, species diversity is found to differ between those who develop mild and severe forms of mucositis [211]. Furthermore, shifts in the oral microbiome during chemotherapy or radiotherapy may allow for the dominance of mucolytic bacteria, particularly *Streptococcus* species that can degrade the protective mucous layer, further predisposing patients to oral mucositis development. The selective inhibition of Gram-negative bacteria, in a placebo-controlled double-blind randomised study by Wijers et al. (2001), was found not to improve radiation-induced mucositis grade [212,213].

Patients who develop the clinical disease of oral candidiasis during radiotherapy or chemotherapy have been shown to harbour increased Candidal load, comprising of *Candida albicans* and *Candida dubliniensis* predominantly, in combination with acid-tolerant bacteria that include *Lactobacillus salivarius, oris* and *crispatus* and *Streptococcus species such as parasanguinis* [162]. A synergistic relationship between *Candida albicans* and *several Streptococcus* species has been suggested [214]. *Streptococcus gordonii* interacts with *Candida albicans*, for example, through its adhesin receptor, improving hyphal development [215] and thus promoting candida tissue invasion. *Candida albicans* remains the most pathogenic species associated with radiotherapy [216].

Finally, in HNC, tumours are often regarded as “cold” or with reduced T cell infiltration, in part due to immune checkpoints, providing cancer protection from the immune system. Pembrolizumab and nivolumab are the commonest anti-PD1 checkpoint inhibitors and are approved by the FDA for the treatment of patients with recurrent or metastatic head and neck squamous cell carcinoma or those with cisplatin resistance. [217,218]. However, there have been patients who respond well to anti PD1/L1 therapy, while others seem to be resistant. Recent evidence now exists to suggest that bacteria may have a potent effect on the successful ability of immune checkpoint inhibitors, which also include the CTLA-4 immunotherapy-type drugs. The species *Akkermansia muciniphila*, in particular, is thought to promote better success with anti-PD-1 therapy by causing the infiltration of CCR9+ CXCR3+ CD4+ T lymphocytes to tumour sites as well as improving the immunosurveillance and preventing the immunosuppression of the host [219,220]. *Ruminococcaceae* were found to be abundant in patients with melanoma responsive to PD-1 therapy, and increased CD4+ and CD8+ T cells, while better cytokine profiles against the melanoma were associated with higher abundances of the *Clostridiales* order, the *Ruminococcaceae* family and the *Faecalibacterium* genus in those patients [221]. In another study, a combination of *Bifidobacterium* and PD-L1 therapy was shown to have an almost complete abolishing effect on solid tumours due to enhanced dendritic cell activity and CD8+ T cell presence in the tumour field [222]. So important is the microbiome on the effect of anti-PD-1 therapy, that a recent clinical trial involved implementing faecal transplantation from responsive donors in anti-PD-1 therapy to recalcitrant cases and has shown promising results in increasing CD8 T cell infiltrations= [223] in tumours in patients that were non-responsive to anti-PD1 therapy.

The effect of the CTLA-4 blockade is now shown to depend on distinct *Bacteroides* species, while *Firmicutes*, in particular *Faecalibacterium*, were associated with an improved clinical response to ipilimumab, by increasing CD8+ cell infiltration to tumours; however, at the same time, this concurred with the increased presentation of a more toxic side effect profile [224]. High levels of the short chain fatty acids butyrate and propionate have also been associated with resistance to the CTLA-4 blockade [225].

## 16. Conclusions

This review highlights the current science linking both the gut and oral microbiome to OSCC aetiopathogenesis and management, but also serves to shed a light on the limitations that exist in this field. Our understanding of the role of the gut and oral microbiome on OSCC development and behaviour is still evolving, but has grown considerably in the last decade. However, it is still difficult to fully impart the true mechanisms by which the oral microbiome may directly lead to OSCC development [226]. Strong reproducible data have now emerged that fully link specific families, genera and species to OSCC aetiopathogenesis; however, there is still room for studies that are longitudinal and that employ increased participant numbers with similar methodologies and less variables. It is imperative that researchers begin to undertake such projects, examples of which have already been employed in other areas of cancer research such as The Cancer Genome Atlas project and the International Genomic Consortium, which provide robust current insights into molecular cancer associations [227]. Longitudinal studies are critical in assessing dynamic and more accurate shifts in the oral microbiome before, during and after OSCC development and can provide key insights into the association of the microbiome with OSCC.
